# Resilience as a mediator in the relationship between ambidextrous leadership and nurses’ positive attitudes towards artificial intelligence

**DOI:** 10.1186/s12912-025-03624-6

**Published:** 2025-08-04

**Authors:** Heba Emad El-Gazar, Eman Sameh AbdElhay, Islam Sameh Abdelhay, Mohamed Ali Zoromba

**Affiliations:** 1 Nursing Administration Department, Faculty of Nursing, Port Said University, Port Said, Egypt; 2 Psychiatric and Mental Health Nursing Department, Faculty of Nursing, Mansoura University, Mansoura, Egypt; 3 Nursing Administration Department, Faculty of Nursing, Mansoura University, Mansoura, Egypt; 4 College of Nursing, Prince Sattam bin Abdulaziz University, Al-Kharj, Saudi Arabia

**Keywords:** Ambidextrous leadership, Positive attitudes towards artificial intelligence, Nurses, Resilience

## Abstract

**Background:**

Leadership plays a pivotal role in adopting new trends within the nursing domain. Yet, the impact of ambidextrous leadership on nurses’ positive attitudes towards artificial intelligence is not well understood. Furthermore, the underlying mechanism governing this relationship has not been thoroughly explored.

**Aim:**

This study aimed to assess the mediating role of resilience in the relationship between ambidextrous leadership and nurses’ positive attitudes towards artificial intelligence.

**Methods:**

A cross-sectional study was conducted among 208 nurses from a university hospital in Egypt. The Ambidextrous Leadership Scale, the Connor Davidson Resilience Scale, and the Positive Attitudes Towards Artificial Intelligence Scale were used for data collection. Structural equation modeling was applied for data analysis.

**Results:**

Resilience partially mediated the relationship between ambidextrous leadership and nurses’ positive attitudes towards artificial intelligence.

**Conclusion:**

Fostering ambidexterity in nurse leaders enhances the resilience of nurses, which in turn, contributes to nurses’ positive attitudes towards artificial intelligence.

**Clinical trial number:**

Not applicable.

## Introduction

Artificial Intelligence (AI), defined as the capability of digital computers to perform tasks traditionally associated with intelligent beings [1], holds significant promise for enhancing the quality and accessibility of medical treatment [2]. AI provides personalized interventions and monitoring with reduced error rates and costs [3]. As a result, healthcare organizations are relying on AI more than ever to facilitate various aspects of patient care and operations [4]. Therefore, understanding the factors that contribute to nurses’ positive attitudes towards AI is crucial for its successful implementation. However, research in nursing that investigates predictors of nurses’ positive attitudes towards AI is still scarce. Although ambidextrous leadership is recognized as crucial for enabling staff to adapt to new market challenges [5], to the best of the author’s knowledge, no studies have yet examined its impact on nurses’ positive attitudes towards AI. Consequently, this study seeks to initially investigate the influence of ambidextrous leadership on nurses’ positive attitudes towards artificial intelligence.

Furthermore, this study aims to explore how ambidextrous leaders sway nurses’ positive attitudes towards artificial intelligence. It builds on ambidextrous leadership theory [6] and the Job Demands-Resources (JD-R) theory [7] and proposes resilience as a mediator in the relationship between ambidextrous leadership and nurses’ positive attitudes towards artificial intelligence. By identifying the mechanisms connecting ambidextrous leadership to these outcomes, we can better understand the processes through which nurses’ positive attitudes towards AI can be enhanced. Consequently, this study seeks to assess the mediating role of resilience in the relationship between ambidextrous leadership and nurses’ positive attitudes towards AI in the workplace.

## Literature review and theoretical foundation

### Ambidextrous leadership

Ambidextrous leadership represents a distinct style wherein leaders exhibit both “opening” behaviors—encouraging exploration, experimentation, and innovation—and “closing” behaviors—fostering efficiency, routines, and implementation among their nursing staff [6]. Prior research has consistently demonstrated that this leadership style yields numerous positive outcomes. For instance, studies have found that it enhances nurses’ psychological safety and creativity [8]. Furthermore, research indicates that nurses working under ambidextrous leaders report higher perceived organizational support, greater knowledge sharing, and stronger commitment [9].

### Ambidextrous leadership and positive attitudes towards AI

Artificial intelligence (AI) refers to machines doing cognitive functions usually related to human minds, such as problem solving, interacting, and learning [10, 11]. AI has become a critical issue in nursing [12]. It has improved care delivery by automating time-intensive tasks that do not require specific nursing skills, allowing nurses to devote more time to direct patient care [3]. AI enhances service quality in terms of efficiency, safety, and healthcare access [13]. Consequently, AI technology has become indispensable in daily healthcare services, potentially reducing the workload of nurses and other healthcare workers and improving work resilience [12].

Ambidextrous leadership theory [6] posits that the flexible switching between opening and closing behaviors of ambidextrous leaders—relying on the situation—facilitates both followers’ exploration and exploitation [14]. Based on this framework, we argue that when nurse managers adopt ambidextrous leadership behaviors, nurses can develop positive attitudes towards AI integration in their hospitals. That is, first, the exploration tendency makes nurses who work with such leaders less resistant to change and treat new technology as more acceptable [11]. Second, the opening behaviors of ambidextrous leaders foster a psychologically safe environment. This encourages nurses to engage with emerging technologies by reducing their fear of making errors during the learning process [8].

Third, ambidextrous leaders who appropriately attend to both exploration and exploitation aspects of their role [6] are considered job resources [15], and according to the JD-R theory, these job resources have the potential to energize individuals. This energy, in turn, motivates individuals to invest in acquiring and utilizing further resources—a process known as a gain spiral [7]. Since AI integration itself represents a valuable job resource that can facilitate nurses’ work and enhance patient care [16], we argue that nurses supported by the resource of ambidextrous leadership will be more motivated to engage with AI. Hence, we hypothesize that:

#### H1

The more that nurse managers exhibit ambidextrous leadership, the more positive the attitudes of their nurses will be towards AI integration.

### The mediating role of resilience

Resilience is a positive adaptive trait that nurses exhibit in the face of adversity. It encompasses three primary qualities: tenacity, strength, and optimism [17]. Strong resilience is associated with effective coping strategies that enhance nurses’ work performance. Moreover, it enables nurses to respond adaptively, enhancing their capability to manage work-related stress, mitigate work fatigue and potential psychological issues, and prevent burnout [18]. Several factors can influence nurses’ resilience, including excessive workload, emotional stress associated with caring for dying patients, issues with patients and their family members, night shift work, and conflicts with managers. These factors may adversely affect performance and reduce life satisfaction [19].

**Fig. 1:**
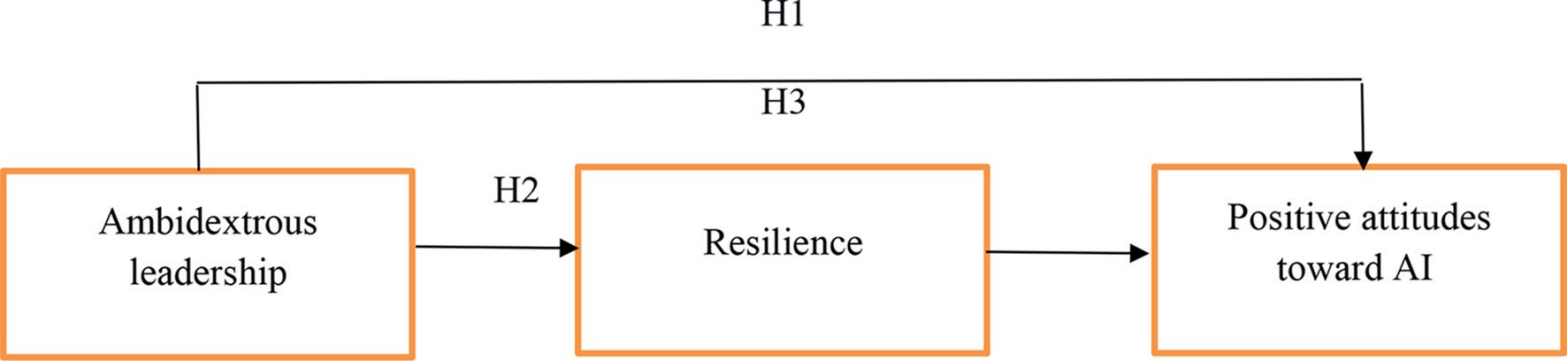
Proposed model

Drawing on insights from ambidextrous leadership theory [6] and the JD-R theory [7], we further proposed the study model that resilience serves as a key mediator in the relationship between ambidextrous leadership and positive attitudes towards AI (Fig. 1). According to ambidextrous leadership theory, leaders adopt “opening” and “closing” behaviors based on situational demands. The leader’s own act of shifting between these two complementary practices is itself a demonstration of resilient behavior [15]. Nurses can, in turn, emulate their leader’s resilient approach, which enhances their own resilience. Additionally, the “opening” behaviors encourage followers to break from rigid work routines [14] and become more flexible, while the communication style of these leaders enables followers to understand and integrate new perspectives [20], further building their resilience.

Within the JD-R framework [7], this resilience is considered a critical personal resource [21]. JD-R theory posits that possessing strong personal resources motivates individuals to enter a “gain spiral,” where they not only protect what they have but also actively seek to acquire new resources [16]. The knowledge and skills required to master a new technology represent such a resource. Therefore, nurses who are energized by their resilience are more motivated to acquire these new skills, which leads them to be more accepting of AI integration in the hospital [22]. Thus, we argue that the ambidextrous leadership of nurse managers has the potential to enhance nurses’ resilience, and this resilience in turn fosters positive attitudes toward AI.

#### H2

The more that nurse managers exhibit ambidextrous leadership, the more psychologically resilient their nurses will be.

#### H3

.Resilience mediates the relationship between ambidextrous leadership and nurses’ positive attitudes toward AI integration.

## The study

### Aim

The study aimed to assess the mediating role of resilience in the relationship between ambidextrous leadership and nurses’ positive attitudes towards AI at work.

## Subjects and methods

### Study design

This is a descriptive, cross-sectional study. The study was reported in accordance with the STROBE checklist.

### Participants and setting

The study population consisted of frontline nurses working at the Main Mansoura University Hospital, Egypt, and were recruited using convenience sampling. The inclusion criteria for the participants were being a registered nurse, having been engaged in clinical nursing work for at least one year, working under the current immediate nurse manager for at least 6 months, and consenting to participate. Nursing students or nurses in leadership positions were excluded from the survey.

Our sample size was estimated using an online a priori sample size calculator for structural equation modeling [23], with power levels (0.95), anticipated effect size (0.3), desired probability (0.05), the number of latent variables (3), and the number of observed variables (36), resulting in a required sample of 184 participants. We oversampled to 350 participants. Out of these, we received 208 valid responses, resulting in an effective response rate of 59.4%.

### Measures

All measures used in this study were translated from English to Arabic employing a back-translation procedure [24]. The translated measures underwent a pilot test with 18 nurses recruited from one of the study hospitals to assess their clarity and comprehensibility. The nurses who participated in the pilot confirmed the clarity and understandability of the measures, and no modifications were made.

#### Demographic form

This form collected data from nurses about their age, gender, marital status, educational level, working department, working years in nursing, and weekly working hours.

#### Ambidextrous leadership

The 14-item Ambidextrous Leadership Scale [6] was used to assess nurses’ perceptions of their nurse managers’ ambidextrous leadership behaviors, comprising two subscales: opening leadership behaviors (7 items) and closing leadership behaviors (7 items). A sample item includes “Gives possibilities for independent thinking and acting.” Participants answered on a 7-point scale, ranging from 1 (strongly disagree) to 7 (strongly agree), where higher scores reflect greater perceptions by nurses of their nurse managers’ ambidextrous leadership behaviors. The findings of the Confirmatory Factor Analysis (CFA) in this study demonstrated a satisfactory fit: χ2/df = 1.83, RMSEA = 0.063, TLI = 0.95, IFI = 0.96, CFI = 0.96.

#### Resilience

The 10-item Connor Davidson Resilience Scale (CD-RISC) [17] was used to assess nurses’ resilience. A sample item includes “Not easily discouraged by failure.” Participants answered on a 5-point scale, ranging from 0 (not true at all) to 4 (true most of the time), where higher scores reflect greater resilience among nurses. The findings of the CFA in this study demonstrated a satisfactory fit: χ2/df = 2.30, RMSEA = 0.079, TLI = 0.94, IFI = 0.96, CFI = 0.96.

#### Positive attitudes towards AI scale

The 12-item Positive Attitudes towards AI Scale [25] was used to assess nurses’ positive attitudes towards AI at work. A sample item includes “I would like to use AI in my own job.” Participants responded on a 5-point scale, ranging from 1 (strongly disagree) to 5 (strongly agree), where higher scores reflect nurses’ more positive attitudes towards AI at work. The findings of the CFA in this study demonstrated a satisfactory fit: χ2/df = 1.93, RMSEA = 0.067, TLI = 0.95, IFI = 0.96, CFI = 0.96.

### Data collection

The data were collected between November 2023 and February 2024. Approval to conduct the study was obtained from the medical and nursing directors of the participating hospital. Subsequently, we presented the study to the wards’ head nurses and gained their permission. Afterward, we distributed paper-and-pencil questionnaires to nurses during their break or meeting times. The first page of the questionnaire provided the background of the study and assured anonymity, confidentiality, and voluntary participation. After the participants completed the questionnaires, they were collected immediately.

### Data analysis

The data were analyzed using SPSS version 27.0 and Amos version 25.0. Descriptive statistics were generated for demographic and main variables. Independent t-test and analysis of variance analysis (ANOVA) were used to investigate the differences in study variables according to sample demographics. Pearson’s correlation analysis was utilized to explore associations among the study variables. The reliability and validity of the study measures were assured. SEM was employed to test the measurement model and to assess the mediating role of resilience in the relationship between ambidextrous leadership and nurses’ positive attitudes towards AI.

### Ethical consideration

The Research Ethics Committee of the Faculty of Nursing at Mansoura University, Egypt, provided ethical clearance for the study. All participants were provided with a comprehensive explanation of the study’s purpose, as well as their right to withdraw. Informed consent was obtained, and participation was entirely voluntary, with confidentiality and anonymity maintained.

## Results

### Participants’ demographics

The demographic characteristics of the participants are presented in Table 1. A total of 208 nurses participated in the study, with a mean age of 34.62 ± 8.70 years. The majority of the participants were female (85.1%), married (80.3%), and held a bachelor’s degree in nursing (36.5%). Among the participants, 46.2% reported working in critical care units, and the average number of years working in the nursing field was 13.68 ± 9.61. Additionally, the majority of the participants (85.6%) reported working less than 45 h per week. There were no significant differences in participants’ demographics across the study variables.

**Table 1:** Participants’ demographics and comparison of the study variables (*N* = 208)

Variable	*n* (%)	Ambidextrous leadership		Resilience		PAAI	
		Mean (SD)	t/F (*P*)	Mean (SD)	t/F (*P*)	Mean (SD)	t/F (*P*)
**Age (years); mean ± SD (34.62 ± 8.70)**							
< 35	111 (53.4)	3.98 (0.87)	t = 1.47 (0.144)	2.13 (0.94)	t = 1.84 (0.067)	3.24 (0.81)	t = 1.08 (0.282)
≥ 35	97 (46.6)	3.79 (1.02)		1.90 (0.88)		3.12 (0.81)	
**Gender**							
Male	31 (14.9)	3.82 (1.15)	t = -0.35 (0.727)	1.85 (1.04)	t = -1.07 (0.292)	3.05 (0.86)	t = 0.92 (0.361)
Female	177 (85.1)	3.90 (0.91)		2.06 (0.89)		3.21 (0.80)	
**Marital status**							
Married	167 (80.3)	3.95 (0.97)	t = -0.15 (0.884)	3.71 (0.84)	t = 1.78 (0.077)	3.49 (0.90)	t = 0.65 (0.452)
Unmarried	41 (18.7)	4.32 (0.88)		3.69 (0.89)		3.53 (0.89)	
**Education**							
Diploma	49 (23.6)	3.98 (0.97)	F = 1.02 (0.385)	1.83 (0.84)	F = 1.09 (0.356)	3.06 (0.98)	F = 1.29 (0.278)
Associate	68 (32.7)	3.94 (0.89)		2.07 (0.95)		3.12 (0.83)	
Bachelor	76 (36.5)	3.74 (1.00)		2.12 (0.94)		3.32 (0.68)	
Graduate	15 (7.2)	4.09 (0.79)		1.94 (0.91)		3.19 (0.72)	
**Working department**							
Medical	32 (15.4)	3.60 (1.23)	F = 1.24 (0.295)	2.05 (1.06)	F = 1.12 (0.346)	3.16 (0.99)	F = 0.54 (0.710)
Surgical	45 (21.6)	3.82 (0.92)		1.85 (0.96)		3.12 (0.66)	
Critical care	96 (46.2)	4.00 (0.82)		2.00 (0.83)		3.17 (0.79)	
Gynecology	13 (6.3)	3.83 (0.81)		2.23 (0.83)		3.19 (0.84)	
Others	22 (10.6)	3.97 (1.01)		2.31 (1.05)		3.42 (0.90)	
**Working Years; mean ± SD (13.68 ± 9.61)**							
< 15	115 (55.3)	3.80 (0.97)	t =- 1.54 (0.126)	2.08 (0.98)	t = 0.99 (0.323)	3.19 (0.84)	t = 0.08 (0.934)
≥ 15	93 (44.7)	4.00 (0.90)		1.96 (0.84)		3.18 (0.78)	
**Weekly working hours**							
< 45	178 (85.6)	3.87 (0.97)	t = -1.07 (0.292)	2.03 (0.92)	t = 0.16 (0.877)	3.19 (0.85)	t = 0.67 (0.503)
≥ 45	30 (14.4)	4.04 (0.78)		2.00 (0.95)		3.11 (0.56)	

PAAI, positive attitudes towards artificial intelligence

### CFA

The study measures were evaluated for their convergent and discriminant validity. Convergent validity was assessed by examining factor loadings, construct reliability (CR), and average variance extracted (AVE) values. For all study measures, items factor loadings ranged from 0.64 to 0.91, surpassing the suggested threshold of 0.5; CR values varied from 0.91 to 0.92, exceeding the standard of 0.7; and AVE values were between 0.51 and 0.53, higher than the minimum acceptable level of 0.5 [26], indicating good convergent validity for the variables. Discriminant validity was assessed by examining HTMT estimates and comparing the square roots of the AVE with the variance shared between constructs. HTMT values ranged from 0.39 to 0.57, falling below the threshold of 0.85 [27], and the square roots of the AVE for all constructs were greater than the inter-construct correlations, confirming good discriminant validity [28]. Additionally, Cronbach’s alphas for all measures ranged from 0.91 to 0.92, surpassing the recommended threshold of 0.70 [29], demonstrating acceptable internal consistency for the measures (Table 2).

**Table 2:** Descriptive statistics, reliability, validity, and the relationships between study variables (*N* = 208)

Variable	Mean ± SD	α	Factor loadings	CR	AVE	1	2	3
1. Ambidextrous leadership	3.89 ± 0.95	0.91	0.67–0.91	0.91	0.53	**0.73**	*0.39*	*0.41*
2. Resilience	2.03 ± 0.92	0.91	0.64–0.78	0.91	0.51	0.36***	**0.71**	*0.57*
3. PAAI	3.18 ± 0.81	0.92	0.66–0.76	0.92	0.51	0.38***	0.52***	**0.71**

AVE, average variance extracted; CR, composite reliability; SD, standard deviation; PAAI, positive attitudes towards artificial intelligence. Bolded values on the diagonals represent the square root of AVE; off-diagonal values are construct correlations; Italics values represent HTMT. ***P < 0.001

### Preliminary analysis

Table 2 presents the means, standard deviations, and correlations among the variables. The average scores for ambidextrous leadership, resilience, and attitudes towards AI were 3.89 (SD = 0.95), 2.03 (SD = 0.92), and 3.18 (SD = 0.81), respectively. These results indicate that the nurses in the study perceived their immediate nurse managers as possessing a moderate level of ambidexterity, while they themselves reported moderate levels of resilience and positive attitudes towards AI. Furthermore, a positive relationship was revealed between ambidextrous leadership and nurses’ resilience (*r* = 0.36, *p* < 0.01), and their attitudes towards AI (*r* = 0.38, *p* < 0.01). Nurses’ resilience was also positively related to their attitudes towards AI (*r* = 0.52, *p* < 0.01).

### Hypotheses testing

**Fig. 2:**
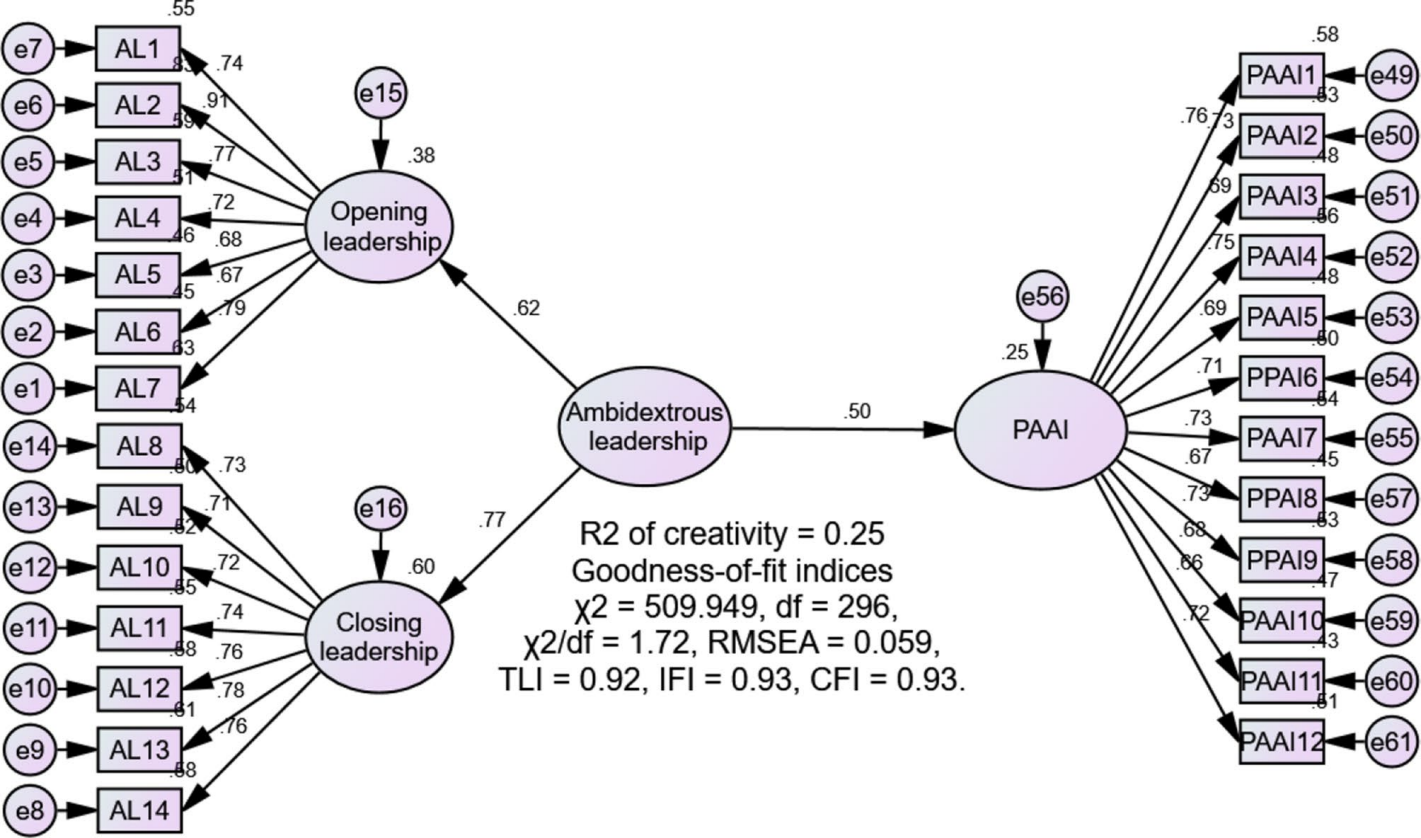
Direct effect model. PAAI, positive attitudes towards artificial intelligence

**Fig. 3:**
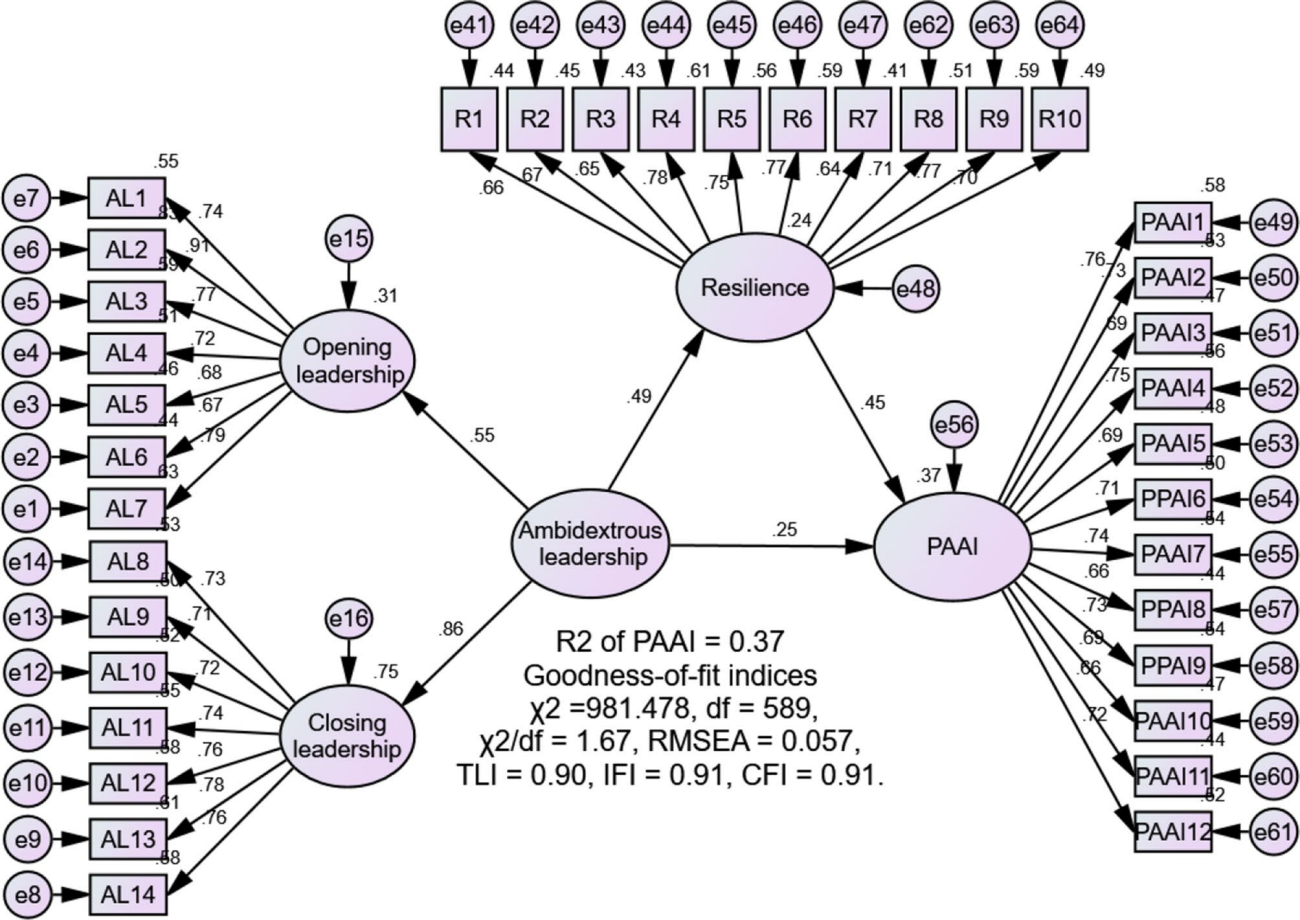
Mediation model. PAAI, positive attitudes towards artificial intelligence

SEM was used to evaluate the study’s hypotheses. Initially, the direct relationship between ambidextrous leadership and nurses’ positive attitudes towards AI was assessed. Results showed that ambidextrous leadership significantly increased nurses’ positive attitudes towards AI (β = 0.50, *p* < 0.001), accounting for 25% of the variance in this outcome. The model fit was satisfactory: χ² = 509.949, df = 296, χ²/df = 1.72, RMSEA = 0.059, TLI = 0.92, IFI = 0.93, CFI = 0.93, supporting H1 (Fig. 2). Next, the mediating role of nurses’ resilience in the relationship between ambidextrous leadership and positive attitudes towards AI was examined using 5000 bootstrap samples and 95% bias-corrected confidence intervals. The model demonstrated an adequate fit: χ² = 981.478, df = 589, χ²/df = 1.67, RMSEA = 0.057, TLI = 0.90, IFI = 0.91, CFI = 0.91. The results indicated that ambidextrous leadership significantly increased nurses’ resilience (β = 0.49, *p* < 0.001), affirming H2. Additionally, resilience significantly increased nurses’ positive attitudes towards AI (β = 0.45, *p* < 0.001). The bootstrapping analysis revealed a significant indirect relationship between ambidextrous leadership and positive attitudes towards AI (β = 0.22, 95% confidence interval: 0.11–0.39). Furthermore, the direct relationship between ambidextrous leadership and positive attitudes towards AI remained significant after including resilience in the model (β = 0.25, *p* = 0.009). These findings suggest that resilience partially mediates the relationship, thereby confirming H3. Ambidextrous leadership and nurses’ resilience accounted for 37.0% of the variance in nurses’ positive attitudes towards AI (Fig. 3; Table 3).

**Table 3:** Effect estimates (*N* = 208)

Path	β	SE	t	*P*	95% confidence interval
					Lower/Upper
Ambidextrous leadership to resilience	0.49	0.15	4.34	< 0.001	0.33/1.05
Resilience to PAAI	0.45	0.09	4.89	< 0.001	0.26/0.68
Ambidextrous leadership to PAAI	0.25	0.12	2.60	0.009	0.01/0.62
Indirect effect of Ambidextrous leadership on PAAI	0.22				0.11/0.39
Total effect	0.47				0.25/0.63

## Discussion

In this study, we assessed the mediating role of resilience in the relationship between ambidextrous leadership and nurses’ positive attitudes towards AI at work. The study hypotheses were empirically supported. The study findings demonstrated that nurse managers’ ambidextrous leadership increased nurses’ positive attitudes towards AI. This is an important result because it suggests the leadership style itself provides a framework for how change is perceived by staff. By creating an environment that is both predictable (due to ‘closing’ behaviors) and open to experimentation (due to ‘opening’ behaviors), these leaders can directly mitigate the anxieties often associated with technological disruption [11]. When nurses feel psychologically safe and view change as manageable, they are more likely to see AI as a valuable tool rather than a threat. The ability of ambidextrous leaders to balance paradoxical behaviors—opening and closing behaviors—facilitates organizational agility and timely responses to market demands [30], such as the adoption of AI. This study corroborates previous research indicating that the practices of nurse managers can improve nurses’ readiness for medical AI [31]. Additionally, prior research has shown that leadership behaviors can decrease AI anxiety [32].

Consistent with the study hypothesis, the findings indicate that nurse managers’ ambidextrous leadership significantly enhances nurses’ resilience. Ambidextrous leaders can flexibly switch between different leadership styles according to situational demands [33]. This behavioral flexibility is crucial, as it creates a psychologically safe environment where nurses feel secure enough to adapt and experiment without fear of failure, a core component of building resilience [8]. As role models to their staff [34], nursing leaders can transmit this flexibility to their teams, thereby enhancing resilience [35]. This process is vital in the high-pressure healthcare setting, where resilience is not just a desirable trait but a necessary buffer against burnout and resistance to constant technological change. These results are similar to those reported by Franken et al. [36], who found that leaders capable of balancing paradoxical behaviors enhance nurses’ resilience.

The study results revealed that increased resilience among nurses enhances their positive attitudes toward artificial intelligence. This is because resilience acts as a cognitive filter [21], reframing the introduction of a complex technology like AI from a potential threat into a manageable challenge. A resilient mindset fosters the confidence and motivation required to engage with, rather than withdraw from, technological innovation [11]. Nurses resilience fosters their ability to positively adopt to diversity and new issues related to patientcare [37]. This is aligning with the “gain spiral” concept within the JD-R theory [7]; possessing the personal resource of resilience builds self-efficacy and energizes nurses to acquire new resources [16], such as the skills needed to master AI. These findings support a previous study that showed resilience and employees’ perceptions of AI are in congruence [38].

Consistent with our hypotheses, the findings indicated that resilience acts as a mediator in the relationship between nurse managers’ ambidextrous leadership and nurses’ positive attitudes toward AI (AI) in the workplace. These findings suggest that ambidextrous leadership, which involves the capacity to flexibly switch between explorative and exploitative behaviors, cultivates an environment that nurtures resilience among nurses. This resilience, in turn, prepares and empowers nurses to adopt a positive attitude toward AI. This crucial finding underscores the vital role of effective leadership and resilience in facilitating the integration of AI into healthcare settings. These findings align with previous studies that demonstrated strengthening bedside nurses’ resilience can be achieved through nurse leader practices [39]. This, in turn, enhances nurses’ readiness to adapt to new issues [40].

### Limitations

This study has several limitations. Firstly, the cross-sectional design necessitates caution in the interpretation of causal relationships. It is recommended that future studies adopt a longitudinal approach to more accurately discern causality. Secondly, the reliance on self-reported data may introduce social desirability bias, which could affect the accuracy of the reported behaviors. This limitation highlights the need for future research to employ alternative data collection methods that may mitigate this bias. Finally, the data collection from a single city in Egypt limits the generalizability of the findings. To enhance the external validity of the results, future studies should consider a more diverse array of sites for data collection.

### Implications for nurses

This study offers several important theoretical contributions. First, it extends the JD-R model by identifying ambidextrous leadership as a specific job resource that initiates a “gain spiral.” We demonstrate that this leadership style builds the crucial personal resource of psychological resilience, which in turn motivates nurses to engage with other potential resources like AI technology. Second, our findings contribute to the leadership literature by situating ambidextrous leadership within the critical context of digital transformation in healthcare. While recent studies, such as the work by Tarsuslu et al., highlight the role of digital leadership in reducing AI anxiety [32], our study adds a complementary and crucial dimension. We demonstrate that it is not only a leader’s technological competency but also their behavioral flexibility and ability to manage paradox that fosters the psychological resilience necessary for nurses to positively embrace AI.

The findings also have significant practical implications for healthcare management and leadership development. Given the importance of enhancing nurses’ positive attitudes towards AI, there is a need to identify factors that could foster such attitudes [32]. Our findings offer two critical practical recommendations for healthcare organizations and nursing management in promoting nurses’ positive attitudes towards AI. First, our results highlight the significant role of ambidextrous leadership in shaping nurses’ attitudes towards artificial intelligence. Healthcare organizations should therefore prioritize the development of ambidextrous leadership among nurse managers, which involves balancing explorative and exploitative behaviors. In the selection process for nurse leaders, organizations should seek candidates who demonstrate the ability to adapt and innovate while effectively managing day-to-day operations. Additionally, healthcare organizations should invest in training programs aimed at enhancing the ambidextrous leadership skills of nurse managers. Given that ambidexterity can be developed [41], such training programs could be instrumental in fostering nurse managers’ ambidexterity, thereby positively influencing nurses’ attitudes towards artificial intelligence.

Second, given that resilience was identified as the key mediating mechanism, organizations should directly invest in building psychological resilience among all nursing staff. This approach complements effective leadership. Practical initiatives could include sponsoring workshops on stress management and mindfulness, establishing formal peer-support groups, and promoting a culture where seeking help is encouraged. By directly strengthening this crucial personal resource, organizations can better equip nurses to handle the pressures of technological change, creating a workforce that is not only well-led but also intrinsically resilient.

## Conclusion

The study provided new perspectives to nursing literature by assessing the mediating role of resilience in the relationship between ambidextrous leadership and nurses’ positive attitudes towards AI at work. This study suggests that nurses’ positive attitudes towards AI can be facilitated by the ambidextrous behaviors of nurse managers. Additionally, the study demonstrated that nurses’ resilience acts as a mediating mechanism in the relationship between the ambidextrous behaviors of nurse managers and nurses’ positive attitudes towards artificial intelligence.

## Data Availability

The datasets used and examined in this investigation are accessible from the corresponding author upon a reasonable request.
